# Development and Modelling of Gallium Nitride Based Lateral Schottky Barrier Diodes with Anode Recesses for mmWave and THz Applications

**DOI:** 10.3390/mi14010002

**Published:** 2022-12-20

**Authors:** Moath Alathbah

**Affiliations:** 1 School of Engineering, Cardiff University, Cardiff CF24 3AA, UK; alathbahm@cardiff.ac.uk; 2 College of Engineering, King Saud University, Riyadh 11451, Saudi Arabia

**Keywords:** GaN diode, RF diode, Schottky diodes, THz applications, GaN semiconductor devices

## Abstract

This paper presents novel multi-channel RF lateral Schottky-barrier diodes (SBDs) based on AlGaN/GaN on low resistivity (LR) (σ = 0.02 Q·cm) silicon substrates. The developed technology offers a reduction of 37% in onset voltage, V_ON_ (from 1.34 to 0.84 V), and 36% in ON-resistance, R_ON_ (1.52 to 0.97 to Ω·mm), as a result of lowering the Schottky barrier height, Φ_n_, when compared to conventional lateral SBDs. No compromise in reverse-breakdown voltage or reverse-bias leakage current performance was observed as both multi-channel and conventional technologies exhibited a V_BV_ of (V_BV_ > 30 V) and an I_R_ of (I_R_ < 38 μA/mm), respectively. Furthermore, a precise small-signal equivalent circuit model was developed and verified for frequencies up to 110 GHz. The fabricated devices exhibited cut-off frequencies of up to 0.6 THz, demonstrating the potential use of lateral AlGaN/GaN SBDs on LR silicon for high-efficiency, high-frequency integrated circuits’ applications. The paper begins with a brief outline of the basic Schottky-contact diode operation. A series resistance analysis of the diode studied in this project is discussed. The small signal equivalent circuit of the Schottky-contact diode is presented. The layout of the diodes studied is described, and their fabrication techniques are briefly mentioned. DC, RF, and low frequency *C*-*V* measurement techniques and measurements to characterize the diodes are outlined. Finally, results and discussions on the effects of multiple recesses under the Schottky-contact (anode) obtained from the *I*-*V* diode characteristics and *C-V* measurements, and the small signal equivalent circuit deduced from RF measurements for different diode configurations, are presented.

## 1. SBD Theory

**Figure 1:**
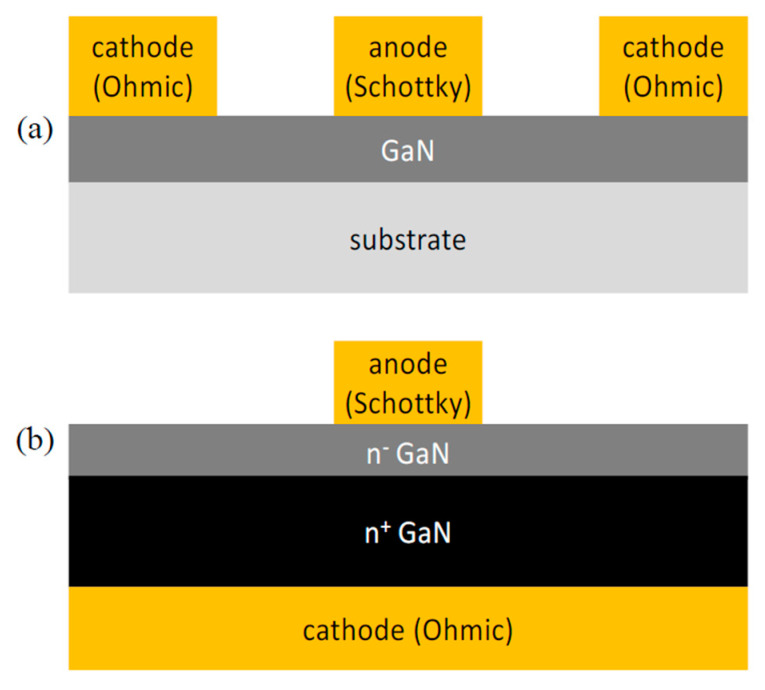
Lateral (**a**) and vertical (**b**) structures GaN-based SBDs.

The GaN-based Schottky barrier diode (SBD) is commonly constructed in three different structures, vertical, quasi-vertical, and lateral, as illustrated in Figure 1. The latter has both contacts, namely the cathode and anode, on the same surface level [1]. Although lateral SBDs are easier to fabricate and test, they suffer from larger barrier height in comparison with vertical ones [2]. However, lateral diodes tend to have a smaller on-resistance and turn-on voltage since current only flows in the drift region, and no substrate loading effects are observed in comparison to vertical diodes. Besides the low on-resistance, lateral SBDs also have a smaller junction capacitance which allow them to be an excellent candidate for high frequency applications.

**Figure 2:**
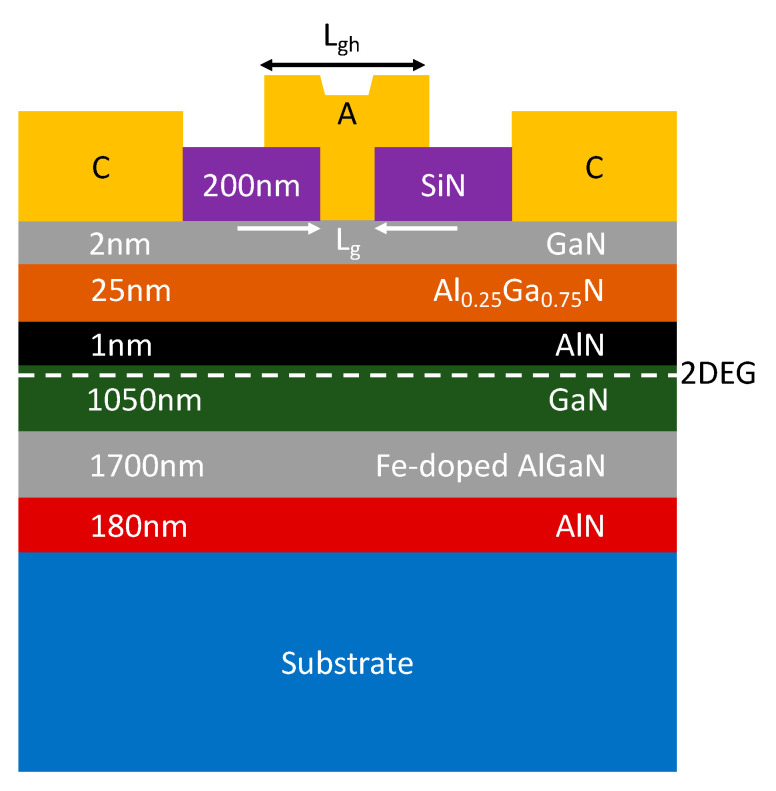
Cross-section view of the fabricated SBDs and the GaN epilayers.

In this project, the ohmic and Schottky contacts are fabricated laterally on the same surface using the wafer demonstrated in Figure 2 to form the AlGaN/GaN heterostructure Schottky diode. The wafer consists of GaN epi-layers placed on a foreign substrate such as Si, sapphire, or SiC. The diode fabrication process starts by forming the isolation, either by mesa or ion implantation using Argon, to remove or damage the active layers between devices in order to self-isolate them for accurate measurements purposes. After that, the ohmic metallic stack-up is deposited and annealed to diffuse the metal into the semiconductor to eliminate the barrier between them and to lower the contact resistance. Next, a passivation layer (SiN) is deposited, then selectively etched for anode and bond-pads’ metal deposition. Finally, a second passivation layer is deposited and selectively etched to expose the measurement pads.

**Figure 3:**
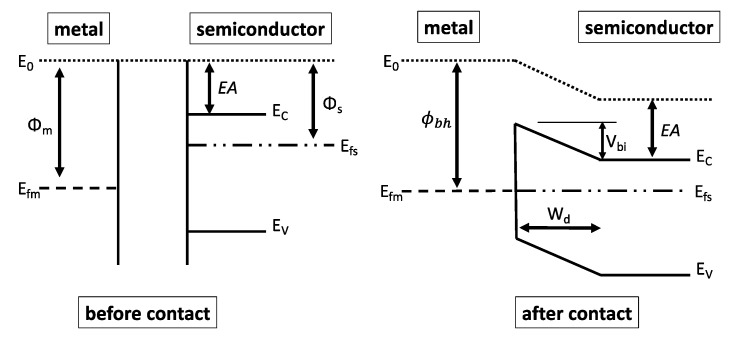
Energy band diagram of a Schottky barrier before and after contact.

Unlike the p-n junction diode, where the current transport is conveyed through the minority carriers, the Schottky diode is a majority-carrier device, which as a result offers a faster switching capability and does not suffer from a charge storage delay resulting in a lower transition time with an instantaneous voltage change across its terminals [3,4]. The Schottky diode is based on a metal-semiconductor contact that forms its anode and ohmic, respectively. The latter (ohmic contacts), such as gallium arsenide, Silicon, or gallium nitride, emits a linear current-voltage (*I*-*V*) relation, where the current is a non-rectified one with a constant conductance value. Schottky (metal such as aluminum, titanium, copper, or gold), on the other hand, is a rectifying-contact formed when the metal (anode) is placed on a semiconductor (cathode) with a different work function, which as a result, creates a barrier height between them producing a nonlinear *I*-*V* curve. Ideally, the Schottky barrier should pass current only under forward bias conditions and block the current flow otherwise (reversed bias). Generally, due to higher mobility, the Schottky diode utilizes an n-type semiconductor (electrons’ majority-carriers) rather than a p-type (holes’ majority-carriers), which results in a higher cutoff frequency and lower series resistance. Figure 3 illustrates the characteristic energy band diagram of a Schottky diode formed between a metal and semiconductor before and after the point of contact, where *Φ_m_* and *Φ_s_* are the energy differences (known as the work function) between the free-space level and fermi levels of the metal and the semiconductor, respectively. When a Schottky contact is generated between the metal and semiconductor, carriers will begin to flow from the semiconductor to the metal to reach a thermodynamic equilibrium condition at which both fermi levels are coincident, and a barrier height is situated between the metal and the semiconductor. This resultant junction potential is called the built-in potential across the diode junction, and it is expressed as:

(1) $$V_{bi}\;=\;\left|\Phi_{m}\;-\;\Phi_{s}\right|$$

The formed depletion region between the metal and the semiconductor is positively charged neutralizing the negatively charged metal, and it imitates the behavior of a capacitor’s dielectric. Assuming that electrons are completely ionized, *n_s_*, which is the sheet density in the active channel, can be considered as the electron concentration, and the width of the depletion region is obtained using Equation (2).

(2) $$W_{d}={\left[\frac{2\varepsilon_{s}\left(V_{bi}-V_{a}\right)}{qn_{s}}\right]}^{1/2}$$

where $\varepsilon_{s}$ = $\varepsilon_{0}\varepsilon_{r}$ GaN dielectric permittivity (GaN ≈ 7.88 × 10^−11^ F/m).

$V_{bi}$ = junction built-in potential

$V_{a}$ = applied voltage

$n_{s}$ = sheet density

q = charge of electron (≈1.6 × 10^−19^ Coulombs)

The charge per unit area $\left(\mathrm{Coulombs}\cdot {\mathrm{cm}}^{-2}\right)$ is given by:

(3) $$Q_{d}=qN_{d}W_{d}={\left[2\varepsilon_{s}\left(V_{bi}-V_{a}\right)\right]}^{1/2}$$

By taking the derivative of charge with respect to the junction voltage, the capacitance of the depletion layer per unit area $\left(F\cdot {\mathrm{cm}}^{-2}\right)$ can be obtained as shown:

(4) $$C\left(V\right)=\frac{\partial Q_{d}}{\partial V}={\left[\frac{\varepsilon_{s}qn_{s}}{2\left(V_{bi}-V_{a}\right)}\right]}^{\frac{1}{2}}=\frac{\varepsilon_{s}}{W_{d}}$$

Knowing the junction capacitance at zero bias $\left(C_{j0}\right)$ and the barrier height $\left(\phi_{bh}\right)$, from measurements, Equation (4) can become:

(5) $$C\left(V\right)=\frac{C_{j0}}{{\left[1-\frac{V_{a}}{V_{bi}}\right]}^{m}}=S^{-1}$$

where *m* = a grading coefficient used as a reflect of the abruptness of the diode junction [5], and its value is usually 0.5 < *m* < 1, depending on the type of device [6].

**Figure 4:**
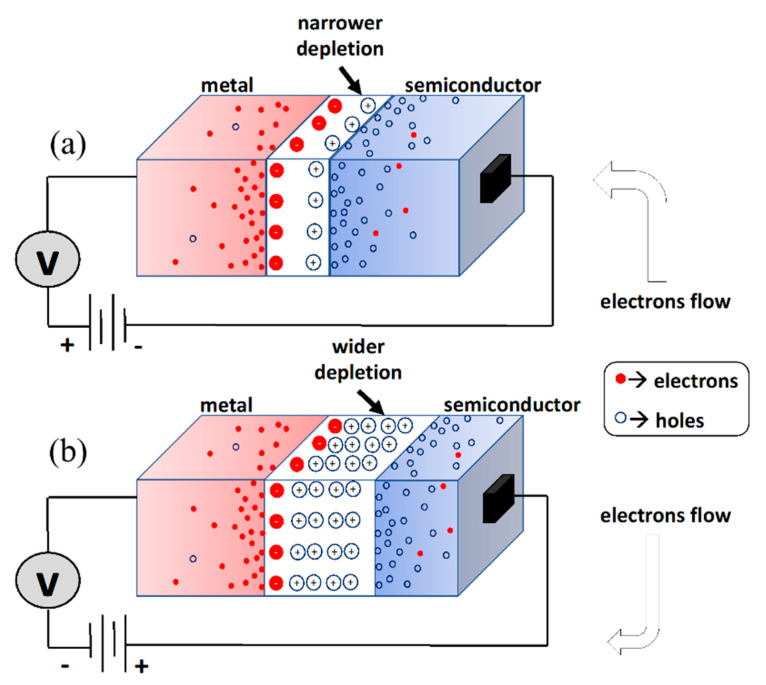
Schottky diode mode of operation as a function of the applied potential, (**a**) forward (varistor) and (**b**) reverse (varactor).

In general, the Schottky diode, under biasing, operates in two modes forward and reverse depending on the applied voltage, and its current is calculated using Equation (6). The applied voltage is utilized to alter the potential barrier magnitude with regard to the desired applications. If a forward operation is required, the potential barrier ($V_{bi}$) is decreased and vice versa for a reverse-operational mode as depicted in Figure 4. On the other hand, at zero-bias, the majority carriers in the Schottky diode require thermal activation to overcome the barrier that resulted from the unequal work function between the metal and semiconductor materials. This type of current transport is known as the thermionic emission, in which the currents flowing from metal to the semiconductor and vice versa are equal in magnitude, and as a result they cancel each other.

(6) $$I_{D}\;=\;I_{S}\left[exp\left(\frac{qV_{a}}{\eta_{n}kT}\right)-1\right]$$

where $I_{S}=the\;saturation\;current=A_{j}A^{*}T^{2}exp\left(\frac{-q\phi_{bh}}{kT}\right)$ and

$A_{j}$ = area of the junction
  $A^{*}$ = Richardson’s constant (GaN ≈ 26 A·cm^2^·K^−2^)
T = absolute temperature
k = Boltzmann’s constant (≈1.38 × 10^−23^ J·K^−1^)
$\phi_{bh}$ = barrier height, which is defined as the difference between the metal work function and the electron affinity (*EA* = the energy required to move an electron from the conduction band to vacuum) of the semiconductor
$\eta_{n}$ = ideality factor; this is a mathematical correction factor added to rectify the diode current nonidealities that arise from practical defects, such as imperfections in the active layer caused by crystal structure damage during the growth process or a contamination within the various fabrication steps affecting the Schottky behavior of the anode. Ideally, its value should be a unity; however, for GaN material, the ideality factor ranges between 1.5 and 2.5 and higher in some cases [7,8,9,10].

## 2. Nonlinearities and Harmonics in SBD

As aforementioned, Schottky diodes are majority-carriers-based devices, which in consequence translates into a negligible delay in their switching-time due to a minimal charge storage or lack thereof. Therefore, the DC transfer function, in Equation (6), of the Schottky diode is also applicable for its AC transfer characteristics. Now, in order to identify the origin of harmonics in diodes, rewriting Equation (6) after an RF signal with a DC component ($V_{dc}$) is inserted to the diode equation $\left[V_{a}=V_{dc}+V_{RF}\cos t\omega_{RF}\right]$. The RF signal has an amplitude $V_{RF}$ and an angular frequency of $\omega_{RF}$; the diode current expression becomes:

(7) $$I_{D}=I_{S}\left[exp\left(\frac{V_{dc}+V_{RF}\cos t\omega_{RF}}{V_{t}}\right)-1\right]$$

where $V_{t}=\eta_{n}kTq^{-1}\approx$ 25mV at room temperature 

By applying Taylor’s series expansion followed by trigonometric double-angle identities [11,12] only to the exponential term that contains the AC component (RF signal) up to the fifth-order term to observe the mathematical basis by which the harmonics are generated, the diode current expression (7) can be rearranged as:

(8) $$\begin{array}{c} I_{D}=I_{S}\left[exp\left(\frac{V_{dc}}{V_{t}}\right)exp\left(\frac{V_{RF}\cos t\omega_{RF}}{V_{t}}\right)-1\right]\;\;\;\\ \;\;=I_{S}\left[exp\left(\frac{V_{dc}}{V_{t}}\right)-1\right]\;\;\;\;\\ \;\;+I_{S}exp\left(\frac{V_{dc}}{V_{t}}\right)\left[\frac{V_{RF}\cos t\omega_{RF}}{V_{t}}+\frac{V_{RF}{}^{2}\cos^{2}t\omega_{RF}}{2V_{t}{}^{2}}+\frac{V_{RF}{}^{3}\cos^{3}t\omega_{RF}}{6V_{t}{}^{3}}\;+\frac{V_{RF}{}^{4}\cos^{4}t\omega_{RF}}{24V_{t}{}^{4}}+\frac{V_{RF}{}^{5}\cos^{5}t\omega_{RF}}{120V_{t}{}^{5}}+\ldots \right]\\ \;\;=I_{S}\left[exp\left(\frac{V_{dc}}{V_{t}}\right)-1\right]\;\to \mathrm{dc}\;\mathrm{current}\;\mathrm{arose}\;\mathrm{from}\;\mathrm{the}\;\mathrm{DC}\;\mathrm{bias}\\ \;\;+I_{S}exp\left(\frac{V_{dc}}{V_{t}}\right)\frac{V_{RF}\cos t\omega_{RF}}{V_{t}}\;\to \mathrm{fundmental}\;\mathrm{RF}\;\mathrm{signal}\;\\ \;\;+I_{S}exp\left(\frac{V_{dc}}{V_{t}}\right)\left[\frac{V_{RF}{}^{2}}{4V_{t}{}^{2}}+\frac{V_{RF}{}^{2}\cos2t\omega_{RF}}{4V_{t}{}^{2}}\right]\to \mathrm{DC}\;\mathrm{and}\;2^{\mathrm{nd}}\;\mathrm{harmonic}\\ \;\;+I_{S}exp\left(\frac{V_{dc}}{V_{t}}\right)\left[\frac{V_{RF}{}^{3}\cos t\omega_{RF}}{8V_{t}{}^{3}}+\frac{V_{RF}{}^{3}\cos3t\omega_{RF}}{24V_{t}{}^{3}}\right]\to \;\mathrm{fundmental}\;\mathrm{and}\;3^{\mathrm{rd}}\;\mathrm{harmonics}\\ \;\;+I_{S}exp\left(\frac{V_{dc}}{V_{t}}\right)\left[\frac{V_{RF}{}^{4}}{64V_{t}{}^{4}}+\frac{V_{RF}{}^{4}\cos2t\omega_{RF}}{48V_{t}{}^{4}}+\frac{V_{RF}{}^{4}\cos4t\omega_{RF}}{192V_{t}{}^{4}}\right]\to \mathrm{DC},\;2^{\mathrm{nd}}\;\mathrm{and}\;4^{\mathrm{th}}\;\mathrm{harmonics}\\ \;\;+I_{S}exp\left(\frac{V_{dc}}{V_{t}}\right)\left[\frac{V_{RF}{}^{5}\cos t\omega_{RF}}{192V_{t}{}^{5}}+\frac{V_{RF}{}^{5}\cos3t\omega_{RF}}{384V_{t}{}^{5}}+\frac{V_{RF}{}^{5}\cos5t\omega_{RF}}{1920V_{t}{}^{5}}\right]\to \;\mathrm{fundmental},\;3^{\mathrm{rd}}\;\mathrm{and}\;5^{\mathrm{th}}\;\mathrm{harmonics}\\ \;\;+\ldots \end{array}$$

From the expression (8), it is apparent that the even-terms nonlinearities in the diode produce an additional rectified dc component beside the preceding even harmonics. The odd terms of nonlinearities, on the other hand, only generate odd harmonics. Furthermore, the dc component is usually utilized in power detection circuitry, and the generated harmonics are exploited in electronic circuits such as mixers and frequency multipliers.

## 3. Series Resistance and Capacitance

**Figure 5:**
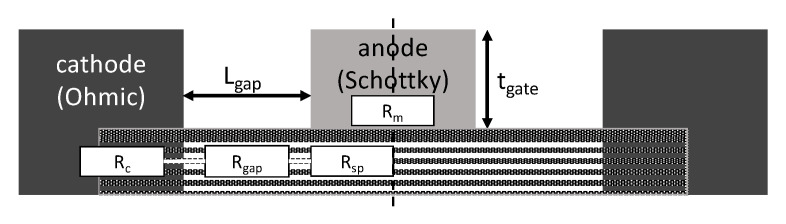
Physical representation of the resistive origin in SBD.

To enhance the diode’s performance, it is crucial to have a low series resistance. Figure 5 illustrates the physical origin of the various components that contribute to the total series resistance of the diode. The diode current expression, which includes the series resistance effect, is given as:

(9) $$I_{D}=I_{s}\left[exp\left(\frac{qV_{a}-IR_{s}}{\eta_{n}kT}\right)-1\right]$$

In general, diode series resistance is comprised of four components: the ohmic contact resistance ($R_{c}$), the resistance of the metallic anode ($R_{m}$), the anode-cathode gap resistance ($R_{gap}$), and the spreading resistance beneath the anode ($R_{sp}$) that includes the aforementioned components that can be expressed as:

(10) $$R_{s}=\frac{R_{m}}{3}+\frac{R_{c}}{2}+R_{gap}+\frac{R_{sp}}{2*3}$$

where $R_{m}$ = anode resistance = $W_{g}{\left[N_{anode}t_{gate}L_{g}\sigma_{m}\right]}^{-1}$

$R_{c}$ = ohmic contact resistance = ${\left[N_{anode}W_{g}\sigma_{c}\right]}^{-1}$

$R_{gap}$ = resistance under the anode-cathode gap = $L_{gap}{\left[N_{anode}W_{g}\sigma_{gap}\right]}^{-1}$

$R_{sp}$ = spreading resistance below the anode = $L_{g}{\left[2N_{anode}W_{g}\sigma_{gap}\right]}^{-1}$

$N_{anode}$= number of anode fingers

$\sigma_{m}$ = conductivity of anode metal

$\sigma_{c}$ = contact conductivity

$\sigma_{gap}$ = conductivity of gap between anode and cathode

$t_{gate}$ = thickness of anode metal

$W_{g}$, $L_{g}$, and $L_{gap}$ are the anode width, anode length, and anode-cathode distance, respectively. The factor 2 is used due to ohmic contact being on both sides of the Schottky. Furthermore, the other factor, 3, is added to make up for the anode metal and the spreading resistances being effective and distrusted by three metal contacts (two ohmic and the anode itself) [13].

However, due to the depletion region being so thin, the Schottky/2DEG diode series resistance, which can be calculated using Equation (11), is largely composed of ohmic contact resistance ($R_{C}$) and the sheet resistance of the semiconductor ($R_{2DEG}$) [14]. The latter is inversely proportional to the electron mobility (${µ}_{n}$) and the electron concentration ($N_{d}$) in the 2DEG. Since the contact resistance arises from the ohmic cathodes on both sides of the anode, a factor of 2 is added to $R_{C}$ [14].

(11) $$R_{s}=2R_{c}+R_{2DEG}\;R_{s}=2{\left[N_{anode}W_{g}\sigma_{c}\right]}^{-1}+\left[L_{gap}-W_{d}\right]{\left[qN_{d}{µ}_{n}W_{g}N_{anode}\right]}^{-1}$$

Knowing the contact specific resistivity from TLM and the sheet resistance value of the semiconductor from wafer characterization using Hall measurements, Equation (11) can be rewritten and further simplified to:

(12) $$R_{s}=\frac{2\rho_{C}+L_{gap}R_{GaN\_sheet}}{N_{anode}W_{g}}$$

The total SBD junction capacitance may be calculated using the expression:

(13) $$C_{j0}=\frac{W_{g}L_{g}\varepsilon_{s}N_{anode}}{W_{d}}$$

Furthermore, in order to predict the diode capacitance accurately, the capacitance per anode finger maybe calculated using this expression [13]:

(14) $$C_{j0}\left(per\;anode\right)=\frac{W_{g}L_{g}\varepsilon_{s}}{W_{d}}\left[1+\frac{2W_{d}}{L_{g}}-\frac{4W_{d}}{L_{g}+2W_{d}}\right]$$

However, to consider the effect of the distance between the cathode and anode, $L_{gap}$, and the Schottky metallic thickness, $t_{gate}$, the capacitance per anode can be given as:

(15) $$C_{j0}\left(per\;anode\right)=2\left(\frac{W_{g}\left(t_{gate}+W_{d}\right)\varepsilon_{s}}{L_{gap}}\right)$$

It is manifested from Equation (15) that the zero-bias junction capacitance of the diode is directly and linearly proportional to the width of the anode. On the other hand, the series resistance is decreasing exponentially with the anode width increase. Therefore, a trade-off is evident between the anode width on one side and the junction capacitance and series resistance on the other side. However, since the anode width and the series resistance have an exponential relation, 10–25 µm is a good trade-off range between the junction capacitance and the series resistance. Although lowering both components has a positive effect on the cutoff frequency of the diode, series resistance is more important to be lowered as it is responsible for the power dissipation in circuits such as frequency multipliers.

## 4. SBD Characterization

The most used figure of merit (FOM) to characterize the varactor diode is the dynamic cutoff frequency, which is a detriment of the maximum operational frequency before the diode is unusable. Practically, the diode actual operational frequency range is usually less than the cutoff frequency, and it can be obtained using the given equation:

(16) $$f_{c}=\frac{S_{max}-S_{min}}{2\pi R_{s}}\approx \frac{S_{max}}{2\pi R_{s}}\;\;\to \;\;assuming\;S_{min}\;is\;negligible$$

where $S_{max}=\frac{1}{C_{min}}$ maximum elastance near breakdown voltage

$S_{min}$ $=\frac{1}{C_{max}}$ minimum elastance at zero-bias 

Another FOM to characterize the varactor diode (which is simply a diode in a reverse-bias mode) is by analyzing its dynamics ($F_{C}$) at the desired operational frequency (output frequency in case of a multiplier), and it is computed using the expression:

(17) $$F_{C}=\frac{S_{max}}{2\pi f_{0}R_{s}}=\frac{f_{c}}{f_{0}}\;$$

where $f_{0}$ = the frequency at which the varactor’s dynamic value is assessed. High $F_{C}$ translates into higher efficiency when designing the frequency multiplier as to be shown in a later section.

Additionally, an important FOM is the capacitance or elastance modulation ratio between the maximum and minimum capacitance, or elastance, at zero and near-breakdown voltages, respectively. In order for the diode to exhibit a high cutoff frequency, the *C_mr_* FOM must be relatively large (>10), which can be calculated using the expression [15]:

(18) $$C_{mr}=\frac{C_{max}}{C_{min}}=\frac{S_{max}}{S_{min}}\;$$

This can be achieved by increasing the number of anodes, which would result in a wider gap between the minimum and maximum capacitance and lower the series resistance. Nevertheless, increasing the number of anodes may have a negative impact on the breakdown voltage and the saturation current.

## 5. Reverse Breakdown Voltage

Breakdown voltage is critical in applications such as frequency multipliers when used in the varactor reverse-bias mode. A higher breakdown voltage contributes to increased output power and conversion efficiency because of a higher ratio between the minimum and maximum elastance of the diode. The breakdown voltage can be calculated using Equation (19), and it can be observed that its value is directly proportional to the critical electric field and inversely proportional to the doping or charge density in the 2DEG [16,17].

(19) $$V_{BV}=\frac{\varepsilon_{s}{\left(E_{c}\right)}^{2}}{2qn_{s}}-V_{bi}\;$$

where $E_{c}$ = the critical electric field (GaN ≈ 3300 kV/cm).

**Figure 6:**
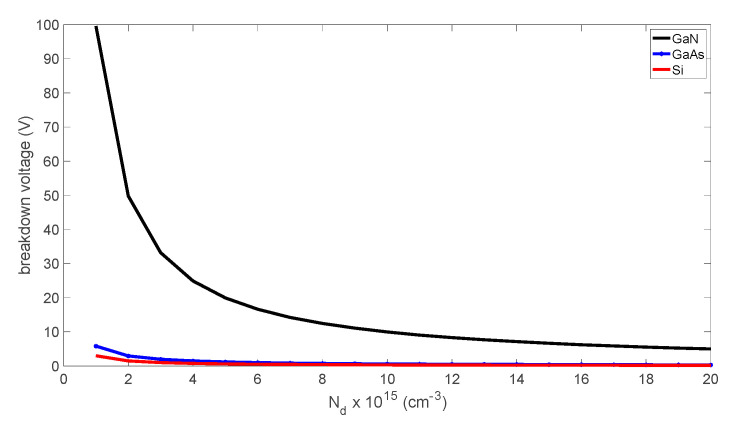
The SBD breakdown voltage as a function of the doping density or electron concentration.

From Figure 6, GaN material exhibited a higher breakdown capability compared to GaAs and Si. Therefore, GaN is clearly superior to other materials regarding its dynamics, computed from (17), as an effect of the breakdown voltage.

## 6. Varactors in Frequency Convertors

A diode-based frequency multiplier is dependent on the nonlinearities of the device. The nonlinearities can be either resistive current-voltage (*I*-*V*) or reactive charge-voltage (*Q*-*V*) relations, varistor and varactor, respectively. In this project, the *Q*-*V* nonlinearities are opted to maximize the efficiency at the expense of operable bandwidth. Theoretically, the available efficiency for the varactor-based multiplier is 100%, whereas the varistor multipliers are limited by a factor of 1/n^2^ where n is the multiplication factor of the frequency multiplier (25% maximum efficiency for a double, for example) [18]. Since a varactor diode topology is chosen, recalling Equation (5), the elastance (*S*) which is the reciprocal of the capacitance can be expressed as [19]:

(20) $$S=S_{0}\left[1+m_{1}e^{j\omega t}+m_{1}^{*}e^{-j\omega t}+m_{2}e^{j2\omega t}+m_{2}^{*}e^{-j2\omega t}+\ldots \right]\;$$

where $m_{1}$ and $m_{2}$, known as the elastance modulation factors, =0.502 and 0.166, respectively, and $S_{0}$ = the elastance at the bias.

Equation (20) assumes that the frequency doubler is ideal, and only the fundamental and the second harmonic are present in the system. The relation between $S_{0}$ and the bias voltage can be displayed as:

(21) $$V_{bias}=V_{bi}-V_{bi}{\left[C_{j0}S_{0}\right]}^{2}\left[1+2{\left|m_{1}\right|}^{2}+2{\left|m_{2}\right|}^{2}\right]$$

Now, the power in the system that is generated by such capacitance nonlinearities which satisfies the aforementioned assumptions can be written as:

(22) $$\left\{\begin{array}{c} P_{system}=4V_{bi}{}^{2}C_{j0}{\left[C_{j0}S_{0}\right]}^{3}\omega {\left|m_{1}\right|}^{2}\left|m_{2}\right|\;\;\\ P_{loss\_{ƒ}_{0}}=4V_{bi}{}^{2}C_{j0}{}^{2}{\left[C_{j0}S_{0}\right]}^{2}\omega^{2}{\left|m_{1}\right|}^{2}R_{s}\;\;\;\\ P_{loss\_2{ƒ}_{0}}=16V_{bi}{}^{2}C_{j0}{}^{2}{\left[C_{j0}S_{0}\right]}^{2}\omega^{2}{\left|m_{2}\right|}^{2}R_{s} \end{array}\right.$$

As a result, the input power at the fundamental (${ƒ}_{0}$), the output power at the second harmonic ($2{ƒ}_{0}$), and the conversion efficiency (η) can be obtained from Equation (23).

(23) $$\left\{\begin{array}{c} P_{in}=P_{system}+P_{loss\_{ƒ}_{0}}+P_{loss\_2{ƒ}_{0}}\\ P_{out}=P_{system}\;\;\;\;\;\;\;\;\;\;\;\;\;\;\;\;\;\;\;\;\;\;\;\;\;\;\;\;\;\;\;\;\;\;\;\\ \eta \;\left(\%\right)=\left[\frac{P_{out}}{P_{in}}\right]\times 100.\;\;\;\;\;\;\;\;\;\;\;\;\;\;\;\;\;\;\;\; \end{array}\right.$$

## 7. Layout and Design

**Figure 7:**
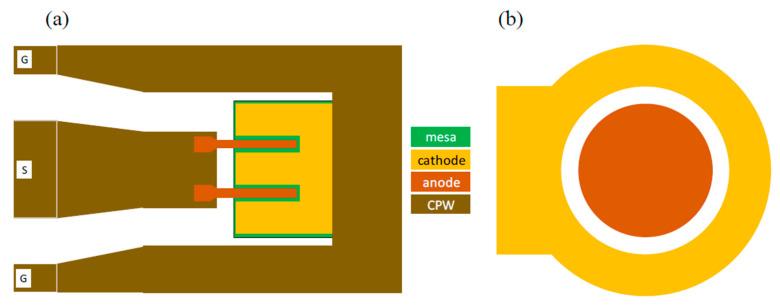
The actual layouts for (**a**) the embedded 2-anode (2-finger) RF SBD in CPW environment and (**b**) large area circular DC diode.

Two different physical layouts of Schottky contact diodes were designed and fabricated during this project. The first type of Schottky contact diode is embedded in a coplanar waveguide structure as shown in Figure 7a. This was used for RF measurements to obtain the small signal equivalent circuit and RF behavior of the diode. The layout of this diode consisted of a Schottky finger (anode) length of 500 nm embedded in a coplanar waveguide structure. In this work, T-shaped cross-section structure devices were used with six and four finger devices with a total anode width of 5 and 10 µm that were realized. The cross-sectional geometry of various structures is shown in Figure 2. Changing the Schottky contact area by changing the Schottky finger length (*L_g_*) and width (*W_g_*) will affect the junction capacitance (*C_j_*). Increasing the number of Schottky contact fingers (*n*) for a constant anode width reduces the series resistance. The separation between ohmic and Schottky contacts was to be 2.0 µm to minimize the series resistance and metal-to-metal capacitance whilst maximizing process reliability and yield. The second type is a large area circular diode (180 µm diameter) Schottky-contact with a 20 µm anode-cathode gap as shown in Figure 7b. This diode structure was used to obtain the *I*-*V* and *C*-*V* characteristics.

## 8. Novel Trenched-Anode SBDs

GaN-based SBDs with low onset voltage (V_ON_), high reverse-breakdown (V_BV_) voltage, and low reverse-current leakage (I_R_), high-switching speed (R_ON_), and high cutoff frequency (ƒ*_c_*) are essentially required to compete with current III-V technologies. Conventional GaN based SBD DC and RF performance is still limited to their large V_ON_, switching loss, and RF leakage when utilizing LR Si substrates. Several researchers recently proposed low V_ON_ along with low I_R_ and high V_BV_ technologies, including a recessed anode, dual-field plates, regrowth cathodes, and a dual-channel field-effect rectifier. However, these approaches require accurate control of anode etching to the 2DEG and a complicated fabrication process, which incorporates reliability issues and extra processing cost. Nevertheless, a 3-D SBDs integrated with a tri-gate MOS structure has shown outstanding DC characteristics at the expense of RF performance owing to the inherently large junction capacitance (*C_j_*) and series resistance (*R_S_*) [20]. Therefore, these techniques are only limited to low-frequency applications. To date, most of the research effort into GaN-based SBDs on silicon is predominantly focused on power electronics, with limited literature targeting RF operation. However, achieving high fc while maintaining low IR and superior V_BV_ remains a challenge. In this work, an optimized multi-channel RF AlGaN/GaN SBDs on LR Si structure is demonstrated using a cost-effective (GaN on LR Si) design which is fully compatible with III-V THz monolithic integrated circuit (THz-MIC) technology. In contrast to conventional SBDs, the newly developed devices significantly enhanced the turn-on characteristics, switching loss, ideality factor (*η_n_*), and ƒ*_c_*, where V_ON_ = 0.84 V, R_ON_ = 0.97 Ω⋅mm, V_BV_ > 30 V, *η_n_* = 1.69, and ƒ*_c_* = 0.6 THz were achieved. This is attributed to the direct contact of the Schottky anode to 2DEG at the sidewalls of the multi-mesa trenches along with proper design geometries to suppress substrate coupling effects.

**Figure 8:**
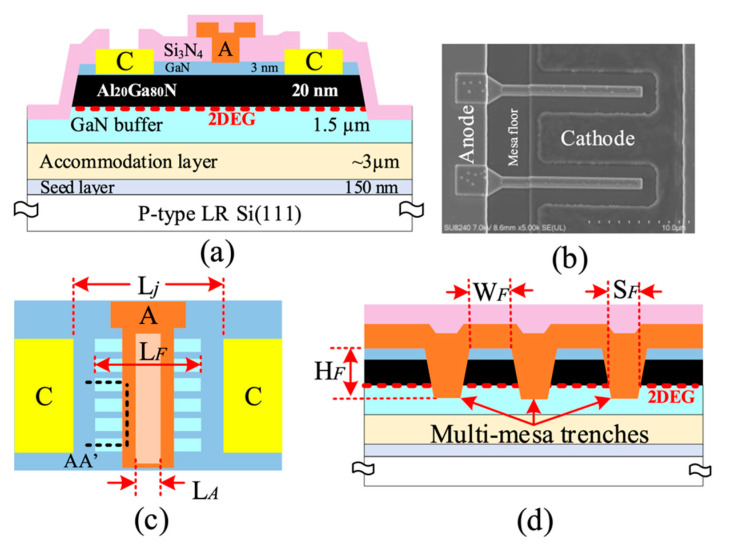
(**a**) Cross-sectional view, (**b**) scanned-electron microscope (SEM) image, and (**c**) top-view of the multi-channel SBDs, and (**d**) Cross-sectional representation of the tri-anode along line AA’ (horizontal to the anode width). Ref. [21] Copyright (2019), with permission from IEEE.

Figure 8 indicates a cross-section of the fabricated AlGaN/GaN SBDs on LR Si using a multi-channel structure, which was simultaneously fabricated with conventional SBDs on the same substrate to allow a precise comparison. A combination of multi-mesa and T-shaped structures was adopted to form the anode to reduce the Schottky barrier height and anode resistivity, respectively. The height (H_F_), width (W_F_), spacing (S_F_), and length (L_F_) of the nanowires were ∼50, 41, 89 nm, and 2 μm, respectively. The Anode length (L_A_) and anode head length (L_AH_) were 0.550 μm and 1.1 μm, respectively, whereas the junction length (L_j_) was 4.28 μm. The total physical anode width was 2 × 10 μm, while the effective anode width for the fin-like anode structure was 2 × 5.83 μm.

The epitaxy material used in this work was grown on LR Si (111) (*ρ* < 40 Ω·cm) provided by Nexperia. The epilayer consists of a 4.65 μm buffer, 20 nm Al_0.2_Ga_0.8_N barrier, and 3 nm GaN cap layer. A sheet carrier density of 5.9 × 10^12^ cm^−2^ and electron mobility of 1713 cm^2^/Vs are determined by using Hall measurements. 

The device fabrication started with defining the Ti/Pt markers, followed by the deposition of Ti/Al/Ni/Au ohmic contacts and rapid thermal annealing at 790 °C in a N_2_ environment to form the cathode. Next, a ∼150 nm depth mesa isolation was performed through Cl_2_/Ar-based inductively coupled plasma (ICP). Then, multi-mesa trenches were defined by e-beam lithography and subsequently etched using Cl_2_/Ar-based ICP with an etch depth of ∼50 nm. A 100 nm Si_3_N_4_ passivation layer was then deposited using a low-stress inductively coupled plasma chemical vapor deposition (ICP-CVD) at room temperature. To form the T-shaped anode, E-beam lithography was used to define anode foot trenches through the Si_3_N_4_ passivation layer using a low damage SF_6_/N_2_ gas mixture reactive-ion etching (RIE), which was followed by Ni/Au metal stack evaporation to finish the T-shaped anode. Windows in the Si_3_N_4_ at the cathode areas were etched prior to the deposition of Ti/Au bond pads and a 160 nm Si_3_N_4_ layer as a final passivation layer. Device fabrication was finalized by Si_3_N_4_ etching in the measurement pad regions. The dimensions of the fabricated devices are outlined in Table 1.

### 8.1. DC Characteristics

**Figure 9:**
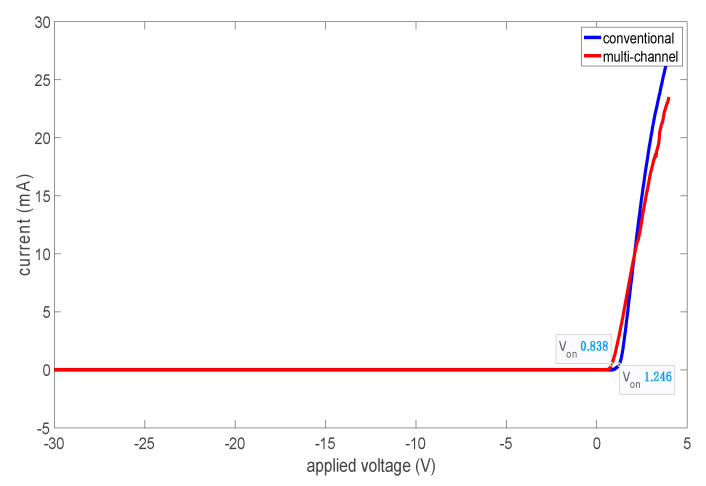
Conventional and multi-channel SBDs *I*-*V* curves.

Figure 9 indicates the typical *I*-*V* characteristics of the fabricated conventional and multi-channel structures at room temperature using a linear scale. The diode current (A/mm) and resistance (Ω⋅mm) of conventional and multi-channel structures are normalized by the total physical anode width (2 × 10 μm) and effective anode width (2 × 5.83 μm), respectively. Figure 9 reveals that incorporating a multi-channel anode structure reduced V_ON_ from 1.246 to 0.84 V together with improved R_ON_ from 1.52 to 0.97 to Ω⋅mm. This is attributed to the direct anode contact to the 2DEG, where the anode is wrapped around the narrow AlGaN/GaN bodies.

**Figure 10:**
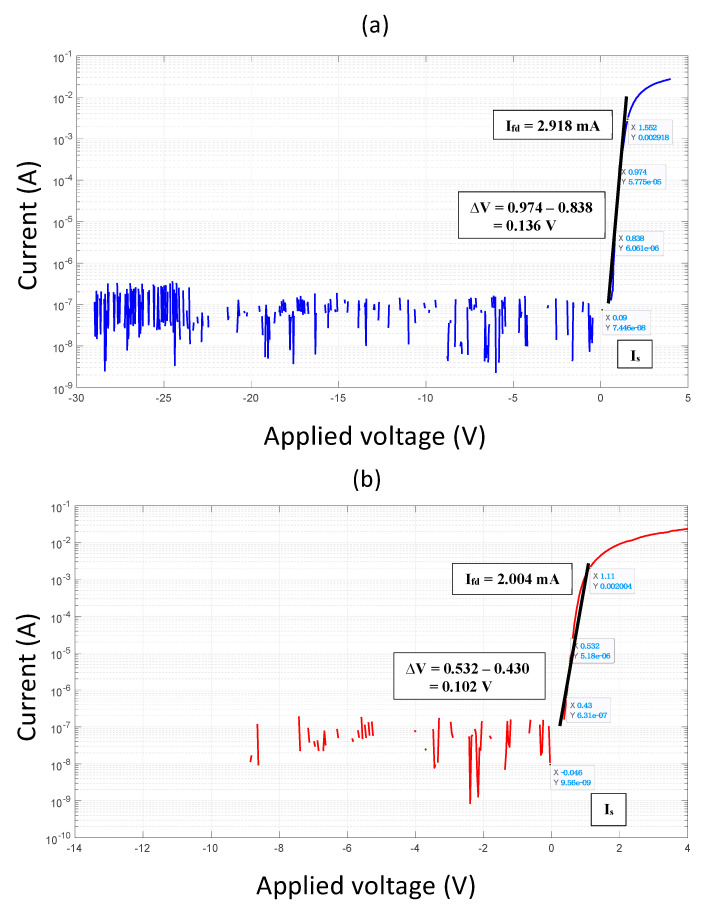
SBDs *I*-*V* semilog, (**a**) conventional and (**b**) multi-channel.

To analyze these findings further, the semilog *I*-*V* plot (shown in Figure 10) is used, which allows the extraction of *η_n_* and *Φ_bh_*. Based on the analytical equations indicated in [8], both device structures exhibited *η_n_* between 1 and 2, indicating the presence of conduction mechanism besides a thermionic emission mechanism. An improvement of 14.28% in *η_n_* (from 1.97 to 1.69) was obtained by the developed multi-channel structure as compared to conventional SBDs. Furthermore, the observed reduction in V_ON_ when using the new structure corresponds to a reduction of 17.5% in n (from 0.78 to 0.64 eV). However, I_R_ was slightly increased with the multi-channel structure, where I_R_ < 38 μA/mm was performed at a reverse voltage of up to 30 V. This is attributed to the additional anode length where the anode is in direct contact to the GaN buffer in the multi-mesa floor regions. The achieved results are comparable to that of SBDs on semi-insulating (SI) SiC with recessed anode and regrowth cathode technologies, with better V_BV_ and I_R_ [2]. This enhancement is mainly attributed to the scale of anode-to-cathode spacing and the use of a T-shaped anode, owing to the reduction in the peak electric field of the Schottky junction.

The following set of equations are used to extract the DC parameters:

(24) $$I_{D}=I_{S}\left[exp\left(\frac{qV}{\eta_{n}kT}\right)\right]$$

rearrangement of the above equation,

(25) $$\frac{I_{1}}{I_{2}}=\left[exp\left(\frac{q\left(V_{1}-V_{2}\right)}{\eta_{n}kT}\right)\right]$$

then,

(26) $$\Delta V=\left(V_{1}-V_{2}\right)=\left[\frac{\eta_{n}kT}{q}\right]\left(\log_{10}\left(e\right)\right)$$

where e = 2.718.

ΔV = the change of voltage which corresponds to the change in current per decade.

Now, the ideality factor is calculated as:

(27) $$\eta_{n}=\left[\frac{q\Delta V}{kT}\right]\left(\log_{10}\left(e\right)\right)$$

In addition, the barrier height is deduced using the expression:

(28) $$\phi_{bh}=\left[\frac{kT}{q}\right]\ln\left[\frac{T^{2}A^{*}}{I_{s}}\right]$$

where $I_{s}$ = the extracted current value from the *I*-*V* semilog plot at the zero-voltage point; k is the Boltzmann constant; T is the diode temperature; and $A^{*}$ is the Richardson constant.

Finally, the series resistance is obtained using equation:

(29) $$R_{s}=\frac{\Delta V}{I_{fd}}$$

where $I_{fd}$ = the first higher current at the point where the voltage, due to the slope decrease caused by the voltage drop across the series resistance, is deviated from the straight fit line of the semilog curve.

**Figure 11:**
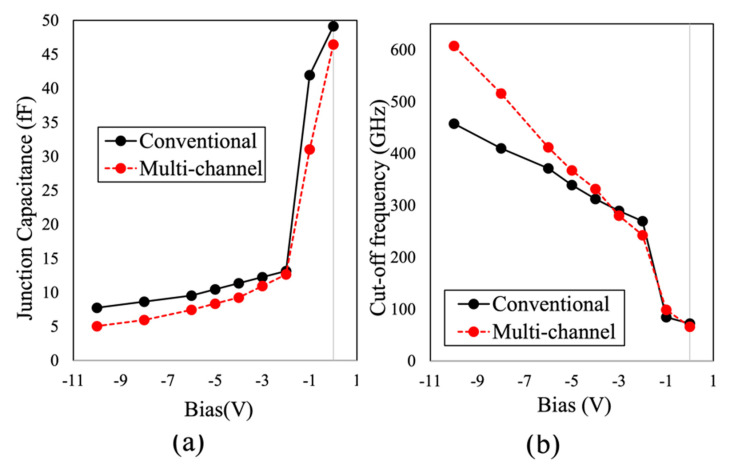
*C*-*V* measurement of the 90 µm diameter circular Schottky diode, (**a**) capacitance and (**b**) corresponding cutoff frequency. Ref. [21] Copyright (2019), with permission from IEEE.

The extracted values of *C_j_* as a function of the applied voltage of the fabricated devices are shown in Figure 11a. *C_j_* was inversely proportional to the applied reverse voltage, where a sharp drop in *C_j_* was obtained when changing the voltage form 0 to −2 V. Furthermore, owing to the direct anode contact to 2DEG for multi-channel SBDs, *C_j_* was significantly reduced at reverse biases beyond −2 V, as compared to conventional SBDs. This reflected a dramatic enhancement in ƒ*_c_* which can be calculated from *R_S_* and *C_j_*. Therefore, ƒ*_c_* was improved by 32.7% (from 457 to 607 GHz), as shown in Figure 11b. However, the achieved ƒ*_c_* of the fabricated lateral SBDs on LR Si is still limited to their larger *R_S_*, which mainly depends on material growth quality and cathode contact resistivity, as compared to SBDs realized on GaN on semi-insulating SiC substrates.

### 8.2. RF Behavior and Small Signal Model

On-wafer small-signal S-parameters’ measurements were performed in the frequency range 0.1 to 110 GHz using an Agilent PNA network analyzer (E8361A) and frequency extenders (N5260A). The system was calibrated with an off-wafer calibration impedance standard substrate (ISS), using a Short-Open-Load-Thru (SOLT) calibration technique. 

**Figure 12:**
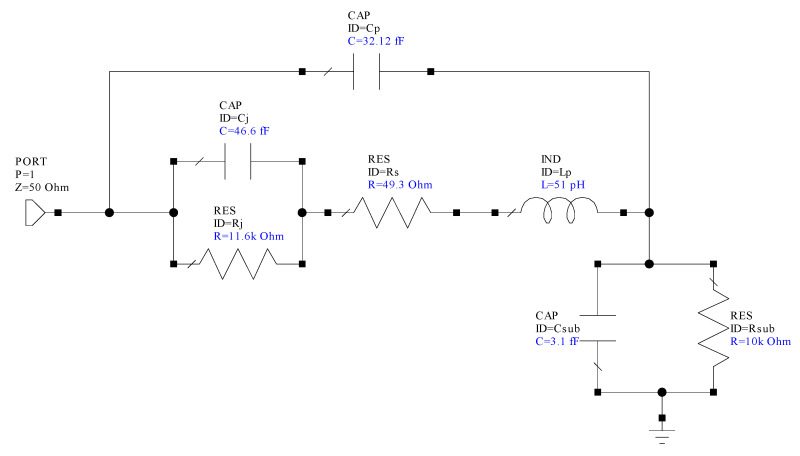
Multi-channel SBD small signal model.

**Figure 13:**
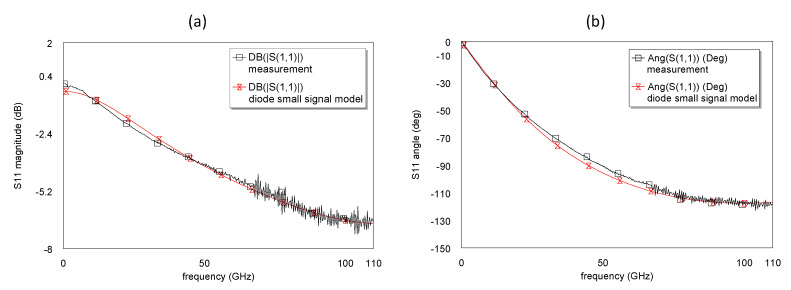
Magnitude (**a**) and phase (**b**) of the return loss, measurement vs. model.

Figure 12 shows the extracted small-signal circuit model of the devices, which was validated by the good agreement between modeled and measured S-parameters up to 110 GHz, as shown in Figure 13. This allows the extraction of SBD intrinsic elements, junction resistance (R_j_), *C_j_*, and *R_s_*, which were used to determine fc of the fabricated devices. As indicated in Figure 12, unlike SI-substrates, substrate parasitic elements (S_sub_ and R_sub_) are incorporated into the standard SBD circuit model when considering lossy Si as a substrate. Furthermore, C_p_ and L_P_ represent pad parasitic components. However, the external parasitic elements have a significant influence on the model at frequencies beyond 20 GHz.

Table 2 shows the extracted circuit element values of conventional and multi-channel structures at no bias (0 V). In contrast to conventional SBDs, an increase in *R_S_* by 15.6% (44.9 to 51.9) and a slight reduction in *C_j_* by 5.5% (from 49.1 to 46.4 fF) were observed for the newly developed fin-type technology. This is attributed to the additional anode length in the multi-mesa trenches and reduction in n, respectively. In addition, the low capacitance value of C_sub_ and high resistance value of R_sub_ (3.1 fF and 10 kΩ, respectively) indicate that the substrate coupling effect could be neglected in both design structures. This was a result of the proper design geometries where the anode-to-cathode separation (2.42 μm) is less than the buffer thickness (4.65 μm).

**Figure 14:**
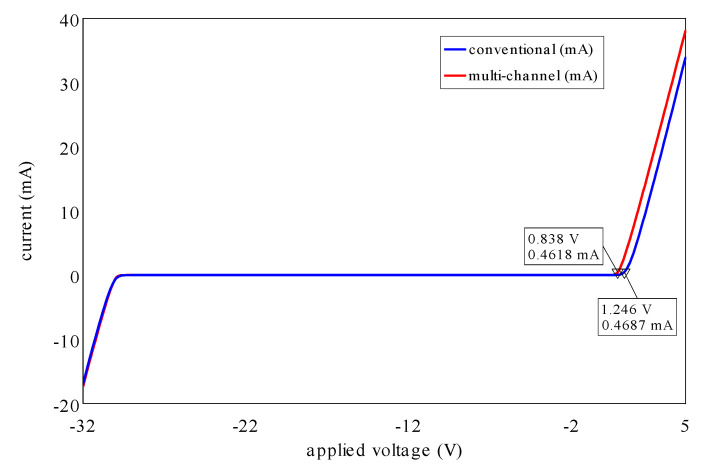
*I*-*V* curves of the conventional and multi-channel SBDs models.

The developed multi-channel RF lateral AlGaN/GaN SBD on LR Si technology was realized in this work. A V_ON_ of 0.84 V along with R_ON_ of 0.97 Ω⋅mm and a *η_n_* of 1.69 were achieved because of the direct Schottky anode contact to the 2DEG resulting in a *Φ_bh_* of 0.64 eV. The fabricated devices exhibited a V_BV_ of greater than 30 V along with an I_R_ of less than 38 μm/mm. In addition, a newly proposed small-signal circuit model was introduced up to 110 GHz. The models are exhibiting similar *I*-*V* characteristics, as shown in Figure 14, which are comparable to the measured results of the devices. A ƒ*_c_* of 0.6 THz at a reverse bias of −10 V was achieved because of the optimized SBD design structure and geometries. These findings enable an effective methodology for the realization of high-performance sub-THz-MIC topologies.

## 9. Summary

In this paper, a novel diode structure was proposed to reduce the barrier height by having a direct contact between the anode and the 2DEG channel. This was achieved by etching trenches below the anode across the channel. This resulted in a clear reduction in the Schottky barrier height from 0.78 to 0.64 initiated by the implantation of the channel trenches. Therefore, the reduction in barrier height led to a decrease in the turn-on voltage and resistance as well. In addition, a newly proposed small-signal circuit model was introduced up to 110 GHz. In addition, a ƒ*_c_* of 0.6 THz at a reverse bias of −10 V was achieved because of the optimized SBD design structure and geometries. These outcomes can be capitalized on to enable an effective approach for the realization of high-performance sub-THz-MIC topologies.

## Figures and Tables

**Table 1:** Dimensions of the fabricated devices.

Dimensions (nm)	L_A_	L_AH_	L_j_	W_g_	H_F_	W_F_	S_F_	L_F_
Conventional	1.1 k	550	4.28 k	10 k				
Multi-channel	1.1 k	550	4.28 k	* 10 k	50	41	89	2 k

* Due to trenches, the effective width of the multi-channel (W_g_) is 5.83 µm.

**Table 2:** The SBD small signal model and *I*-*V* extracted key parameters.

Parameters	*C_j_* (fF)	*R_s_* (Ω)	*I_s_* (A)	*η_n_*	*Φ_bh_* (eV)	V_BV_ (V)
**Conventional (2-anode)**	48.3	44.6	7.45 × 10^−8^	1.97	0.78	−30
**Multi-channel (2-anode)**	46.4	49.3	9.56 × 10^−9^	1.69	0.64	−30

## Data Availability

The data presented within this paper are available on a reasonable request from the author.
